# Barriers to sexual reproductive health services and rights among young people in Mtwara district, Tanzania: a qualitative study

**Published:** 2012-12-26

**Authors:** Rita Moses Mbeba, Martin Sem Mkuye, Grace Elias Magembe, William Lubazi Yotham, Alfred obeidy Mellah, Serafina Baptist Mkuwa

**Affiliations:** 1 AMREF in Tanzania, Dar es Salaam, Tanzania

**Keywords:** Youth friendly services, reproductive health, health services, family planning, counseling and testing

## Abstract

**Background:**

In Tanzania over 1/3 of the population is under 24 years. Nationwide 23% of teenagers have started childbearing. However, Mtwara Region has the highest percentage (25.5%) of teenagers who begin childbearing early. Mtwara District has a teenage pregnancy rate of 11% with young people utilizing sexual reproductive health services (SRHS) less frequently than adults.This study aimed at gaining insights on barriers to the utilization of SRHS in Mtwara district.

**Methods:**

A qualitative study was carried out using focus group discussions, facility assessment interviews and case studies. A total of nine focus group discussions (comprising 8 to10 persons per group) were conducted among girls (10-18 years), community leaders and adults. Data was transcribed using pattern matching methods then merged into relevant themes for analysis and interpretation.

**Results:**

The study revealed that a good number of health facilities do not have skilled service providers (SPs) on sexual reproductive health rights. Girls start sexual intercourse between 9 and12 years. Services sought included; education, family planning and voluntary counseling and testing. However, the services were inaccessible due to lack of privacy, confidentiality, equipments and negative attitudes from SPs. Initiation ceremonies, early marriages and gender disparities were mentioned as social-cultural barriers to SRH rights.

**Conclusion:**

This study has demonstrated that factors such as lack of youth friendly services, gender disparity and unfavorable socio-cultural practices may create barriers to accessing adolescent SRHS and rights. Therefore, there is a need to integrate youth friendly services in health facilities and advocate for behavior change.

## Background

A youth is an individual within a period of transition from childhood to adulthood, between the age of 10-24. Worldwide the terms “youth”, “adolescent” and “young people” are interchangeably used, often meaning the same thing but occasionally differentiated. During this period youth undergo biological transition, attain reproductive capability and psychological transition [1–4]. According to WHO, one in every five people worldwide is an adolescent (10-19years). It is estimated that there are about 1.2 billion adolescents worldwide. An estimate of 1.7 million youth lose their lives due to accidents, pregnancy related complications and violence [5].

In Tanzania young people (under the age of 24) comprise 32% of the population[6] These young people face many significant sexual reproductive health challenges such as limited access to youth friendly services including information on growth, sexuality and family planning. This has led youth into risky sexual behaviors resulting to high STI and HIV prevalence, early pregnancy and vulnerability to delivery complications resulting in high rates of death and disability [7–9]. The National Demographic survey of 2010, shows that Maternal mortality ratio (MMR) is still high at 454/100,000 live births. A total of 23% of women aged 15-19 have started childbearing, while 44% of them were either mothers or are pregnant with their first child by the age of 19 [6].

Mtwara is among the regions in the Southern part of Tanzania with the highest percentage (25.5%) of teenagers who begin childbearing at young age [6]. Mtwara region presents a higher total fertility rate (TFR) of 6.1 compared to the national TFR of 5.4 children per woman's life. Mtwara district council is one of the six districts in Mtwara region with a total population of 204,157 [10]. The district is divided into 28 wards with a total of 38 health facilities. Young people (10-24) compose 28% of the district population. The district has illiteracy rate of 25%. According to the district health department records of 2010, the MMR is 238/100,000 live births while teenage pregnancy rate is 11% [11].

In Mtwara district, socio-cultural factors underpin negative gender inequities in the home and wider community, leading to disparities in access to, and quality of health, educational, financial and other services. Traditional practices such as early, forced marriages and initiation ceremonies are some of the factors which some studies have found contributing to teenage pregnancy in the Region [12].

The voice of the youth project which is implemented by African Medical and Research Foundation (AMREF) aims at empowering young girls in Mtwara region to demand, access and utilize quality sexual reproductive health services [13]. National SRH policy of 2003, explicitly states that adolescence stage is an optimal critical time to ensure access to reproductive health information and services so as to enhance healthy life styles [14]. Therefore, AMREF through *Voice of youth* project conducted a baseline assessment aiming at establishing baseline status of project indicators and targets as well as gaining insights on barriers to youth SRH rights and services in Mtwara district.

## Methods

### Study design

A qualitative study was carried out through focus group discussions, facility assessment interviews and case studies. The design ensured representation of ideas, opinions, suggestions and recommendations concerning sexual reproductive health and rights from community to district level. A total of nine focus group discussions (composing 8 to10 persons per group) were conducted among girls (10-18years), community leaders and adults.

### Setting

The study was conducted in Mtwara district council in Mtwara Region. The district is administratively divided into 6 divisions, 28 wards 157 villages, 38 health facilities, 118 primary schools and 20 secondary schools. The district covers an area of 3,597 square kilometers and has a population estimate of 204,157. Young people (aged 10 - 20 years) are estimated to be 28%. The ethnic groups are Makonde, Makua and Yao. Economic activities include farming of cashew nuts, cassava and sorghum [15].

The baseline study was conducted in 12 (43%) wards which were randomly selected. The wards included; Chawi, Kiromba, Kitere, Madimba, Mahurunga, Mayanga, Mpapura, Nanguruwe, Nanyamba, Naumbu, Tangazo and Ziwani ward. A total of 13 (34%) health facilities were sampled using purposive sampling. The health facilities selected for study were located within the project area (in the 12 wards). The criteria used for selecting the health facilities were catchment area covered by the facility, type of facility and location within the 12 wards (Table 1).

**Table 1:** List of health facilities versus services offered

FACILITY NAME	TYPE OF FACILITY	SRHS OFFERED										Selected for the survey
		VCT	CTC	STI	PMTCT	HBC	PICT	PEP KITS	FP	Condom Promotion & provision	Care during child birth	
**Chawi**	Disp	V		V	V				V	V	V	V
**Dihimba**	Disp	V	V		V			V	V	V	V	
**Hinju**	Disp	V			V				V	V	V	
**Kiromba**	Disp	V	V		V			V	V	V	V	V
**Kitaya**	Disp	V	V	V	V	V		V	V	V	V	
**Kitere**	HC	V	V	V	V	V	V	V	V	V	V	V
**Kiyanga**	Disp				V				V	V	V	
**Lobobe**	Disp				V				V	V	V	V
**Lipwidi**	Disp				V				V	V	V	
**Madimba**	Disp	V	V	V	V	V	V	V	V	V	V	V
**Mahurunga**	HC	V	V	V	V	V	V	V	V	V	V	V
**Mangopachanne**	Disp				V				V	V	V	
**Mbawala**	Disp	V	V	V	V	V	V	V	V	V	V	
**Mbembaleo**	Disp				V				V	V	V	
**Mgao**	Disp				V	V			V	V	V	
**Mkunwa**	Disp				V	V	V		V	V	V	V
**Mkutimango**	Disp				V				V	V	V	
**Mnima**	Disp	V		V	V				V	V	V	
**Mnongodi**	Disp				V				V	V	V	
**Mnyawi**	Disp				V				V	V	V	
**Mparura**	Disp	V	V	V	V	V	V	V	V	V	V	V
**Msangamkuu**	Disp	V	V	V	V		V	V	V	V	V	
**Msimbati**	Disp	V	V	V	V			V	V	V	V	
**Mtimbwilimbwi**	Disp	V			V				V	V	V	
**Mtiniko**	Disp				V				V	V	V	
**Muungano**	Disp				V				V	V	V	
**Nalingu**	Disp				V				V	V	V	
**Namgogoli**	Disp	V			V				V	V	V	
**Namisangi**	Disp				V				V	V	V	
**Namtumbuka**	Disp	V		V	V	V	V		V	V	V	
**Nanguruwe**	HC	V	V	V	V	V	V	V	V	V	V	V
**Nanyamba**	HC	V	V	V	V	V	V	V	V	V	V	V
**Naumbu**	Disp	V	V	V	V	V		V	V	V	V	V
**Nitekela**	Disp			V	V				V	V	V	
**Njengwa**	Disp			V	V				V	V	V	
**Nyundo**	Disp				V				V	V	V	
**Tangazo**	Disp	V			V				V	V	V	V
**Ziwani**	Disp				V				V	V	V	V
**TOTAL**	20	13	16	38	12	10	13	38	38	38		

V – service available/offered

### Study population

A total of 9 focus group discussions (composed of 8-10 persons per group) were conducted with girls, adults and community leaders. Furthermore, a case study and facility assessment interviews among service providers were conducted in the 13 HFs to assess the suitability and capacity needs of the HFs to provide quality sexual reproductive health services.

### Data sources

Three focused group discussions were conducted from the selected wards with girls aged 10-18 years. These groups contained in and out of school girls (separated primary school girls from secondary school girls). The groups had the following characteristics; similar age and sex, comparable levels of education, spoke the same language and were from similar socio-economic backgrounds. Discussions were held with separate focus groups of unmarried and married adolescent girls. The number of participants per group ranged from 8 to 10 persons. This number was preferred since it was manageable and small enough giving everyone the opportunity to express his or her opinion.

FGD with community leaders, influential figures and adults: Six focus group discussions with community leaders and adults (men and women separately) were conducted. The discussions focused on Adolescent sexual reproductive health knowledge, attitude and practices (KAP) as well as factors hindering access to ASRHS and rights. The discussions also included action taken to address ASRH issues, support provided, challenges and experiences. The study conducted a total of nine focus group discussions because different constituency groups needed to be included in the research.

A total of 3 case studies were documented during the study. This paper reports on one of the case studies since it was focusing on enforcement of sexual reproductive health rights at community level which is the main item addressed by the project.

Facility assessment interviews were done in 13 health facilities using key informant interview guide. This guide was also used to interview the district officials. A purposeful sampling was used to identify types of respondents at facility, community, school and district level. The idea was to have the sample representation of key informants and project beneficiaries (young girls). It was also agreed that, respondents should be representatives from the selected catchment areas. This was done under the supervision of the study moderator.

### Data collection

Data collection tools were pre-tested before finalization for actual data collection. The tools used include focus group discussion guide and key informant interview guide. The actual data collection was done by research assistants under close supervision by the hired moderator. Research assistants (who had health professional backgrounds) were oriented on the study design, data collection tools, objectives and processes before embarking on field data collection. During data collection data was recorded using a digital recorder and files downloads into a laptop the same day for transcription, coding and analysis. The transcriptions were done by the hired consultant and an assistant data analyst. The study team obtained permission to conduct the research from the District authorities. Throughout data collection, interviewees were asked for consent before the interview began. For under 18 interviewees, adults and community leaders were consulted to permit the interviews. Written consent was also obtained for the photos that could be used in the final report. Confidentiality was maintained by assuring study participant's names are not used during data collection so the study used initials.

### Data quality assurance

Data quality and consistency was taken care of throughout the process. Quality was ensured by recruitment of qualified interviewers and direct participation of the consultants during field work. Effective training of interviewers was conducted to ensure detailed understanding of objectives, process, and output requirements for consistency and completeness. Close supervision of fieldwork by consultant also ensured that interviewers collect data coherently and in a manner that maintained data integrity and completeness. The tools used were translated from English to Swahili Language by a language professional then were pre-tested to check its accuracy before collection of data. The information was transcribed by interviewers and revised by the consultant on daily basis.

### Data analysis

Data was transcribed using pattern matching methods. It was merged into relevant themes derived from study objectives for analysis and interpretation.

## Results

### District capacity on youth friendly services

**Figure 1:**
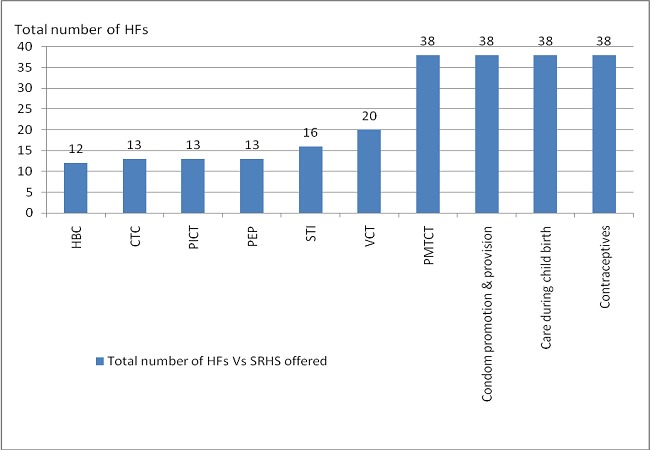
Sexual Reproductive Health Services Provided In Health Facilities (Hfs) In Mtwara District Council Hf – Health Facilities, Srhs- Sexual Reproductive Health Services, Hbc-Home Based Care, Ctc - Care And Treatment Centers, Pict- Provider Initiated Counseling And Testing, Pep – Post Exposure Prophylaxis, Sti – Sexually Transmitted Infections, Vct – Voluntary Counseling And Testing, Pmtct- Prevention Of Mother To Child Transmission

The findings show that, none of the 38 facilities in Mtwara district has designated areas for provision of youth friendly services (YFS) since services provided were adult centered. SRH services offered included; condom provision, contraceptives, PMTCT and postnatal care (Figure 1). Findings also showed limited number of service providers with SRHR skills. Facilities were found lacking privacy, bed examination screen, Information materials, screens and curtains. Additionally, district records showed that only 20 HSPs are trained on ASRHS.

### Knowledge on sexual reproductive health

The findings showed that most young girls age 10 - 18 years in Mtwara rural district, do not have a place within their communities where they are able to visit and talk about relationships, sex, contraception, sexually transmitted infections and HIV/AIDS. However, nearly every girl stated to have started sexual intercourse between the age of 9 and 12. For example a response from a girl at Nanguruwe ward was as follows; *“I started sexual intercourse when I was 10 years old.”* Another one stated that: *“I have had sex since when I was 9 years old”*.

### Attitude on sexual reproductive health

The community members and service providers in the district think it is inappropriate for girls of age 10 - 18 to access SRHS especially the family planning. Stigma and discrimination to SRHS was also reported and confirmed by the adults and community members in FGDs. For example one adult male at Kiromba ward stated that; *“I do not think if its right for young people to use family planning methods since it will affect their reproductive system and unable them to get children”*.

### Practices on SRH

#### Sexual abuse

A case story of a girl from Mtiniko primary school is a vivid example on sexual abuse occurring in Mtwara district as presented by the case story below.

Sexual abuse by relatives; a case story of a girl at Mtiniko Primary school.

A case of a school girl raped by an uncle was reported to the teacher, by the victim following implementation of UNICEF “Tuseme” project activities. The girl claimed that, for a long period she had been repeatedly raped by her uncle who claimed to do so as compensation for costs he incurs to feed and educate her. Her mother was married and moved to her husband, so the girl had to stay with her uncle.

The teacher reported the case to the district authority. Several days passed without the school or the district authorities taking any actions. After several days of consultations, the girl was advised to continue having sex with her uncle with purpose of collecting evidence on the event. However, the girl couldn't implement the advice then the district authority advised the girl to go stay with her mother. Until completion of the baseline data collection exercise, there was no psychological support provided to the girl, nether a follow up taken on the case or the uncle.

#### Commercial sex

Girls who stated that they had started having sexual intercourse, reported that they did sex either in exchange for money or food. Muna is a 14 years old from one of the project target wards. She told the interviewer that, her first partner had to buy for her a blouse to put on during Maulid day before she could agree to have sex with him.

#### Community social-cultural practices

The findings show responses from adults, youth and community leaders on several community social cultural aspects, knowledge, attitude and practices that negatively affect adolescent sexual reproductive health services and rights. They include:

Misconception about suitability of contraceptives to young people particularly the girls. Adults and religious leaders at Mahurunga, Mayanga, Nanguruwe and Mpapura wards stated *“if young girls use family planning especially the injectables, it negatively affect their reproductive abilities in the future”*

Misconception about use of condom due to unpleasant sexual experiences so men and women do not prefer using them. A girl stated “My partner does not agree to use condoms since he believes it reduce sexual flavor”

Extended *Unyago* ceremonies: The *unyago* ceremony is a traditionally initiation ceremony for girls in the region. The training is done by special experienced teachers’ renowned in the communities. Participants stated that *“Unyago is also regarded as prestigious event and sign of success for parents especially the mothers”*.

Video watching at local night clubs: Business men in the communities use generators to run video watching night clubs for a fee between 500 and 700 Tsh. The kind of videos shown are on pornography and sexual relationships.

#### By-laws responsive to SRH

Furthermore, actions stated to be taken by community structures are to setting and enforcing local bylaws that protect rights of youth. For example a community leader stated:*“We have set a bylaw that video watching should be 3 days per week from 4.00 pm to 7.00pm.”* However, the enforcement of this bylaw is poor. In addition another community leader stated that they have set lawful measures taken for men impregnating/sexually abusing young girls: *“We have set a bylaw which states that any man who impregnates/ sexually abuses a young girl will be reported to the lawful bodies for further punishment since most of them escape and no actions are taken”*.

## Discussion

Findings revealed low knowledge on ASRH right among health service providers. Moreover, many people consider them the best sources of such information thus being major influence on public's sexual reproductive health [16]. Tanzania's national ASRH guideline demands proper and quality ASRHS, that all adolescents have to be informed on their SRH rights, such rights should be made known first to service providers and significant others [17–19]. This shows a high concern of promoting for SRH information in schools, homes and communities to prevent unprotected sexual practices among youth. Most girls reported starting sex between the age of 9 and 12 indicating a younger age compared to findings from Europe and central Asia where girls start sex for the first time between the age of 15 and 19 [20]. In most developing countries, young people have inadequate access to appropriate sexual reproductive health information contributing to unprotected sexual practices leading to unwanted pregnancies, HIV/AIDS and STIs [21–23]. There is a need for introducing SRH interventions focused on health education. Therefore, contribute to changing attitude towards youth when seeking SRH services [24].

Presence of service providers skilled on youth friendly services is one thing but supply of equipments required for enabling the provision of these services is another vital requirement which enables providers to deliver quality services. This was among the challenges that this study revealed; whereby facilities lack privacy, confidentiality, record keeping ledger and waiting benches. However, the national guideline for ASRH further demand that service delivery points should be organized in a manner that is conducive for provision of adolescents friendly reproductive health services [19, 25]. This study shows that a good number of facilities providing SRH services although most of these services are adult centered. Several studies show the need for integrating youth friendly service in health facilities considering the set standards using the available resources in our localities, enabling youth being free to disclose sensitive matters on SRH to health providers [22, 26].

This study found most facilities lacking privacy, not having staff of the same sex, having health workers with negative attitudes, stigma and discrimination, providers lacking confidentiality. In several studies, confidentiality has been found to be a contributing factor associated with utilization of sexual reproductive health services among youth [22, 24, 27]. Studies done in Botswana found out that most health providers have negative attitude towards youth when seeking SRH services [28, 29]. Staff need to be trained on principles such as confidentiality and privacy, this helps improve utilization of health services among youth [30]. Youth friendly service provision needs to be integrated in the district since these skills will change health providers’ attitude towards youth when seeking health services. Youth friendly services are provided to young people to meet their needs in an environment that attracts their rights in utilizing the services [31, 32]. These services can be offered within the context of health facilities or community settings. However, a study done in Uganda found that more adolescents significantly utilized SRH services when provided through mobile services as compared to stationed facilities [33].

A case study finding found sexual abuse, assault and harassment as one of the problem occurring at community level. This is mostly because people do not know their rights and some consider it a shame upon the family hence it is kept secret. A study done in Tanzania found that in cases of sexual abuse the victims have no knowledge of their rights. They do not know the Tanzania child law of 2009 which states “it's the responsibility of any community member aware of child abuse to report the incident to the authority that can help the child. While Section III, cap 94 - 96 says, the ward or government has responsibility to protect the well being of children. Thus, communities need to be made aware of these laws and the responsible bodies should take actions [34, 35]. Furthermore, in this study girls stated practicing prostitution in exchange for money, cloth or food, thus, increasing the risk of unintended teenage pregnancies. Similar findings were reported in Uganda whereby many girls reported verbal, physical harassment and sex in exchange of money as part of their daily lives [36].

Cultural practices contribute to the utilization level of sexual reproductive health services in societies. This study found that factors such as initiation ceremonies (*Unyago* and *Jando* - is a local rite of passage into social roles and sexuality), misconception on the use of contraceptives among young people, early marriages, stigma and discrimination of young mothers discouraging them from returning back to school after delivery. This is a principle method that relays social messages of self upkeep, community building, women roles and responsibilities. However it goes beyond coaching young girls how to satisfy male partners. Boys are trained and expected to practice sex as a sign of masculinity and maturity. These teachings should be sought out according to the age of the girl. *Unyago* is often singled out as a key cause of early pregnancy although there are other forces larger the *unyago* that determine sexuality among adolescents in Mtwara [12]. However, in African societies there are other cultural practices such as female genital mutilation resulting to delivery complications among women. It is a custom that requires a girl to be circumcised and married at a young age. This tradition is commonly practiced in nomadic settings found in Kenya, Ethiopia and Tanzania [37].

Early marriage is commonly practiced in Tanzania whereby recent data shows more than 23% girls between the age of 15 and 19 get married [4, 6]. This is also experienced in Nepal, whereby most parents prefer their daughters to get married early and bear children within the first year after marriage. Most parents were comfortable for their children to access and use condoms because they believed they would be protected from both unwanted pregnancies and sexually transmitted diseases. However, few adults and religious leaders believed that if young girls use family planning methods especially the injectables (depo provera), it will negatively affects their reproductive abilities in the future. Furthermore, in Nepal, use of family planning methods is prohibited among unmarried young couples but acceptable to married couples. This promotes higher chances of unsafe sexual practices among youth [38].

In addition, the study reveals that video shows on ponography and sexual relationships were found to be among contributing factors leading youth to engage in risky sexual behaviors. Video show are normally shown in local night clubs thus encouraging conducive environment for sexual activities for various groups including putting the girls into a danger of rape. This is against the Tanzania child law of 2009 which says, the owners of bar, disco, or night clubs are prohibited to allow a child to enter the premises. Likewise in Nepal growth of communication channels and transport networks has created different social cultural environments which is conducive to social interactions leading to teenage pregnancy, STI and HIV/AIDS [22]. In addition, exposure to western culture through TV and Internet tends to encourage initiation of sexual intercourse among adolescents in several countries [39–41]. However, if communication channels are appropriately used they tend to help increase awareness, acceptability and utilization of SRH services among community members [42].

Study finding established several actions taken by the community structures and district officials including introduction of by-laws addressing SRH services and rights. The actions should be highly promoted to ensure community members understand their importance. For example communities should set bylaws such as taking legal action against all people curtailing girls from school. However this has not been a success, as community members collaborate with the perpetrators and settle the matters privately; normally by promising to take care of the child after birth or marry the girl. This contributes to early marriages and thus high illiteracy among women. Given that the Marriage Act still allows marriages at 15 if consented by parents [43].

Further studies need to investigate the relationship between socio-cultural factors and utilization of reproductive health services. This will enable determination of significant associations of predictive variables leading to barriers to SRH services.

### Limitations

This study extends the current level of knowledge, attitude and practice as well as barriers to access to SRH services among youth. However, since the study relied on self-reported information, information bias is likely to have occurred due to over or under reporting. Qualitative data provided a more detailed explanation about the current situation. Moreover, quantitative methods that focuses on the associations of various variables needs to be done.

## Conclusion

This study has demonstrated challenges, needs and opportunities awaiting the district. Factors such as lack of SRH information, absence of skills for YFS, lack of equipments for provision of YFS, unfavorable cultural practices, gender disparities, poor enforcement of by- laws may create barriers to access to SRH services for youth. Therefore, there is a need of integrating youth friendly services in health facilities and advocate for behavior change at community level. Consequently, it is envisioned that findings from this assessment will enrich project implementation strategies, district planning and positively inform the district management teams on ASRH programming and decision-making. Considering these findings, the following recommendations might assist when setting strategies in relation to SRHS: improve HFs supply of equipments and tools, training SPs on YFS, distribute YFS information materials, revamp HFs mechanisms to enhance community support and promote enforcement of youth SRH related bylaws.
